# LHRH sparing therapy in patients with chemotherapy-naïve, mCRPC treated with abiraterone acetate plus prednisone: results of the randomized phase II SPARE trial

**DOI:** 10.1038/s41391-022-00533-6

**Published:** 2022-04-16

**Authors:** Carsten-Henning Ohlmann, Michelle Jäschke, Peter Jaehnig, Susanne Krege, Jürgen Gschwend, Heidrun Rexer, Kerstin Junker, Roger Zillmann, Christoph Rüssel, Eva Hellmis, Henrik Suttmann, Martin Janssen, Jan Marin, Andreas Hübner, Michael Mathers, Jochen Gleißner, Michael Scheffler, Susan Feyerabend, Jens Telle, Jörg Klier, Michael Stöckle

**Affiliations:** 1 Department of Urology, Saarland University Medical Center, Homburg/Saar, Germany; 2 Department of Urology, Johanniter-Kliniken Bonn, Bonn, Germany; 3 pj statistics, Berlin, Germany; 4 Department of Urology, Evangelische Kliniken Essen Mitte, Essen, Germany; 5 Department of Urology, Klinikum Rechts der Isar, Technical University Munich, Munich, Germany; 6 Meckevidence, Schwarz, Germany; 7 Urologie Pankow, Berlin, Germany; 8 Urologie Borken, Borken, Germany; 9 Urologicum-Duisburg Fachärztesozietät, Duisburg, Germany; 10 Urologikum Hamburg MVW, Hamburg, Germany; 11 Department of Urology, University Münster, Münster, Germany; 12 Urologie Kempen, Münster, Germany; 13 Zentrum für Onkologie und Urologie, Rostock, Germany; 14 Pandamed, Remscheid, Germany; 15 Uro-Gyn-Praxis Am Wall, Wuppertal, Germany; 16 Urologische Gemeinschaftspraxis, Zwickau, Germany; 17 Studienpraxis Urologie, Nürtingen, Germany; 18 Urologische Praxisgemeinschaft, Wolfsburg, Germany; 19 Urologische Partnerschaft, Köln, Germany

**Keywords:** Prostate cancer, Cancer therapy

## Abstract

**Background:**

Although the benefit of androgen deprivation therapy (ADT) continuation in metastatic castration-resistant prostate cancer (mCRPC) remains controversial, clinical evidence is lacking. Recent results indicated that treatment with abiraterone acetate (AA) plus prednisone (P) further suppresses serum testosterone levels over ADT alone, suggesting that continuation of ADT in the treatment of mCRPC may not be necessary.

**Methods:**

In this exploratory phase 2 study, mCRPC patients were randomized with a 1:1 ratio to receive either continued ADT plus AA + P (Arm A) or AA + P alone (Arm B). The primary endpoint was the rate of radiographic progression-free survival (rPFS) at month 12. Secondary endpoints included PSA-response rate, objective response, time to PSA progression and safety.

**Results:**

A total of 68 patients were equally randomized between the two study arms. Median testosterone-levels remained below castrate-levels throughout treatment in all patients. According to the intention-to-treat analysis the rPFS rate was 0.84 in Arm A and 0.89 in Arm B. Moderate and severe treatment-emergent adverse events were reported for 72% of the patients in Arm A and for 85% of the patients in Arm B.

**Conclusions:**

AA + P treatment without ADT may be effective in mCRPC patients and ADT may not be necessary in patients receiving AA + P.

## Introduction

Prostate cancer (PC) is the second most common cancer diagnosed in men and the fifth leading cause of death worldwide [1]. PC is typically castration-sensitive at the time of initial diagnosis, but the majority of patients undergoing androgen deprivation therapy (ADT) develop castration-resistant PC (CRPC) [2]. Changes in the normal androgen receptor signaling pathway are considered crucial for CRPC pathogenesis [3].

Abiraterone acetate (AA) is a prodrug of abiraterone, which is a selective and irreversible inhibitor of 17α-hydroxylase/C17,20-lyase (cytochrome P450C17[CYP17]), a key enzyme in testosterone synthesis [4]. AA plus prednisone (P) is approved in Europe in combination with LHRH therapy for the treatment of metastatic, castration-resistant prostate cancer (mCRPC) upon pre-treatment with docetaxel, based on the results of the COU-AA-301 study [5] and for the treatment of chemotherapy-naïve asymptomatic or mildly symptomatic mCRPC patients based on the results of the COU-AA 302 study [6].

Treatment of mCRPC patients with AA + P leads to an increase in median survival compared to P alone [7, 8]. Results from the COU-AA-302 study revealed that progression-free survival (PFS) was significantly improved by treatment with AA + P [9] and overall survival (OS) was prolonged compared with P [6]. In castrated patients receiving LHRH-therapy and treated with AA + P testosterone further decreased to undetectable levels [10] suggesting that AA + P may induce and maintain testosterone deprivation without concomitant LHRH therapy.

Although AA + P may maintain androgen deprivation in the absence of LHRH-therapy, treatment without concomitant LHRH-therapy may impair efficacy of AA + P due to androgen deprivation independent effects. Cessation of LHRH-therapy may lead to rapid recovery of luteinizing hormone (LH) levels, even after long-term medical castration [11, 12]. LH may also act directly on PC cells via LH-specific receptors inducing expression of key steroidogenic enzymes [13] and thus enhancing rescue pathways of testosterone and dihydrotestosterone production [14].

Whether continuation of LHRH therapy in patients with CRPC should be mandatory is debatable and guidelines recommend continuation of LHRH-therapy [15] despite potential side effects and avoidable expenses [16]. Currently, clinical trials are being conducted to investigate the efficacy of AA in different clinical settings (summarized in [16]). The results of the LATITUDE trial [7, 17], showing that the combination of AA + P with ADT in CSPC was associated with significantly longer OS than placebo plus ADT while maintaining a manageable safety profile, further increased the medical need to evaluate the efficacy of AA + P treatment while sparing LHRH-therapy. SPARE was the first study designed to investigate the efficacy of AA + P with and without concomitant LHRH-therapy in patients with mCRPC.

## Patients and methods

A detailed description of the SPARE study protocol was previously published [18]. The study protocol was approved by the relevant institutional review boards and independent ethics committees.

### Patients

Adult, male patients with histologically or cytologically confirmed adenocarcinoma of the prostate, documented metastatic disease, documented PC progression, asymptomatic or mildly symptomatic from PC, medically castrated (testosterone levels of <0.5 ng/ml), were eligible for inclusion in the study. Exclusion criteria included surgical castration, symptomatic mCRPC (BPI ≥ 3), opioid pain medication, liver or visceral metastasis, and known brain metastasis. Prolonged effects of LHRH-analogues were controlled by in- and exclusion criteria (see Supplementary Table 1).

### Trial design and procedures

This was an exploratory Phase 2 multi-center, randomized, open-label study with a randomization allocation ratio of 1:1 [AA + P + LHRH-therapy (Arm A) versus AA + P (Arm B)]. For both groups, patients received a dose of 1000 mg AA and 10 mg P daily. AA was administered as 4 × 250-mg tablets and P was administered as 5 mg orally twice a day (BID). Patients randomized to the LHRH-therapy group received the same LHRH-therapy as prior to enrolment.

The study was conducted between May 2014 and March 2018 at 22 study sites, of which 12 recruited patients, in Germany. Study objectives and endpoints are listed in Table 1. Efficacy assessments included computed tomography and bone-scans every 3 months with disease assessment by the investigator, and PSA levels every 4 weeks. Levels of serum androgens and hormones were analyzed centrally monthly within the first 3 months and every 3 months thereafter. Safety was monitored by documented information on concomitant medication, adverse events (AEs), physical examination, body weight, ECG, cardiac ECHO, and vital signs. Adverse events were coded using the MedDRA coding system and all AEs were graded according to the National Cancer Institute Common Terminology Criteria for Adverse Events, version 4.03 (NCI-CTCAE, June 14, 2010). Treatment emergent adverse events (TEAEs) were defined as those events that occurred or worsened on or after first dose of study drug up through 30 days post last dose.

**Table 1:** Objectives and endpoints of SPARE study.

Primary objective
• To analyze the clinical benefit of abiraterone acetate plus prednisone while sparing LHRH-therapy in chemotherapy-naïve patients with metastatic castration-resistant prostate cancer (CRPC).
Secondary objectives
• To establish additional clinically relevant information regarding early PSA responses to abiraterone and to correlate these with radiographic-progression free survival.
• To investigate effects of both treatment arms on hormones of the pituitary gonadal axis.
• To characterize the safety profile of abiraterone acetate while sparing LHRH-therapy in comparison to continuing LHRH-therapy.
Primary efficacy endpoint
• Rate of radiographic progression-free survival (rPFS) at month 12*
Secondary efficacy assessments
• PSA response rate scored in patients achieving a post-treatment PSA decline of at least 50% according to the protocol specific PCWG2 criteria.
• Time to PSA-progression measured from the time interval from the date of randomization to the date of the PSA progression as defined in the protocol-specific PCWG2 criteria. The determination of PSA progression requires that the patient receive at least 3 cycles of therapy.
• Objective response rate in patients with measurable disease (RECIST).
• Value of the bone-scan index as a biomarker of response to treatment.
• Changes in pituitary gonadal axis by measurement of androgens and hormones (LHRH, LH, FSH, testosterone, DHT).
Safety assessments
• Medical history, vital sign measurements, physical examination, and body weight Concomitant therapy and procedures.
• Adverse events (AEs) and serious adverse events (SAEs), including laboratory test AEs will be graded and summarized according to the National Cancer Institute (NCI) Common Terminology Criteria for Adverse Events (CTCAE), version 4.03.
• Blood chemistry, hematology, coagulation studies, serum lipids, and urinalysis.

### Statistical analysis

Patient disposition and efficacy analyses were performed on data from the intention-to-treat (ITT) population (equivalent to Full Analysis Set [FAS]). All patients who received any part of abiraterone acetate were included in the analysis of safety (safety population). Evaluation of an eligible and/or per-protocol population was not specified in the protocol. The baseline measurement was the last value on or before the date of first study treatment. All statistical analyses were performed using SAS 9.4 and SPSS 24.0.

This was an exploratory phase 2 trial where neither the primary endpoint radiographic progression-free survival (rPFS) nor the secondary endpoints were powered for statistical significance (all results are to be interpreted in the exploratory sense). Each treatment arm should include 30 patients evaluable for the primary endpoint. Assuming a drop-out rate of 15% in each arm it was estimated that 70 patients needed to be recruited for this trial.

## Results

### Patients and treatment

In total, between May 2014 and March 2018, 89 patients were screened at 12 study sites in Germany and 68 patients were randomly assigned 1:1 (web-based, block of 6) to one of the two study arms (Supplementary Fig. 1); therefore, both arms comprised 34 patients. Of these, 4 (Arm A) and 3 (Arm B) patients did not meet the inclusion criteria and were included by mistake. Two patients in Arm A and one patient allocated to Arm B did not receive study medication. The study was completed according to protocol by 26 patients allocated to Arm A and 21 patients allocated to Arm B. Median treatment duration of the drop-out patients was 93 (Arm A) and 394 (Arm B) days, however, in both arms 6/34 (17.6%) patients dropped out within 12 months of treatment. Treatment compliance, assured by regular drug accountability every 4-weeks during the regular visits of the patients, was good as only 0.1% of AA doses were missed in each Arm. The intention-to-treat population comprised a total of 68 patients (Arm A = 34 patients and Arm B = 34 patients) and the safety population comprised a total of 65 patients (Arm A = 32 patients and Arm B = 33 patients).

Baseline characteristics were balanced between study arms (Table 2). Briefly, median age in Arm A was 74 years (ranging from 60 to 83 years) and in Arm B 76 years (ranging from 60 to 86 years). All patients had previously received hormonal therapy. Median serum testosterone level at baseline was 0.08 ng/ml in Arm A and 0.06 ng/ml in Arm B. Median PSA level at baseline was 31.9 ng/ml in Arm A and 19.5 ng/ml in Arm B. 20 patients (59%) in Arm A and 15 patients (44%) in Arm B had measurable disease (including primary) at baseline.

**Table 2:** Demographic and patient characteristics (ITT population). The data are presented as *n* (%) unless otherwise noted.

	Arm A (*N* = 34)	Arm B (*N* = 34)
Age (years)		
Median	74	76
Range	60–83	60–86
ECOG		
0	26 (76)	28 (82)
1	7 (21)	6 (18)
2	1 (3)	0 (0)
Gleason score at diagnosis		
5	1 (3)	1 (3)
6	3 (9)	4 (12)
7	11 (32)	9 (27)
8	7 (21)	8 (23)
9	8 (23)	8 (23)
10	1 (3)	1 (3)
Previous cancer therapy		
Prior prostatectomy	17 (50)	10 (29)
Prior radiotherapy	15 (44)	14 (42)
Hormonal therapy	34 (100)	34 (100)
Hormone therapy		
Leuprorelin	16 (47)	18 (53)
Goserelin	0 (0)	2 (6)
Buserelin	6 (18)	3 (9)
Pamorelin	3 (9)	4 (12)
Degarelix	4 (12)	0 (0)
Abarelix	0 (0)	0 (0)
Unknown	5 (15)	7 (21)
Time last LHRH to treatment start (days)		
* N*	31	33
Median	106	97
Range	31–1318	16–270
Serum testosterone level at baseline (ng/ml)		
* N*	34	33
Median	0.08	0.06
Range	0.029–4.210^a^	0.029–0.429
Metastases at baseline		
Bone only	14 (41)	15 (44)
Lymph node/soft tissue	3 (9)	3 (9)
Bone and Lymph node/soft tissue/prostate	16 (47)	15 (44)
No metastases	1 (3)	1 (3)
Measurable disease (including primary)		
Yes	20 (59)	15 (44)
No	14 (41)	19 (56)
Baseline BPI-score		
0–1	29 (85)	29 (85)
2–3	3 (9)	5 (15)
>3	2 (6)	0 (0.0)
Analgetic use at baseline		
No	27 (79)	23 (68)
Yes	7 (21)	11 (32)
Baseline hemoglobin level (g/dl)		
* N*	34	34
Median	13.1	13.6
Range	8.6–15.5	10.6–15.5
Baseline LDH (IU/L)		
* N*	30	32
Median	223.5	223.2
Range	126–635	150–550
Baseline alkaline phosphatase (IU/L)		
* N*	33	32
Median	118	87.2
Range	48–319	36–1625
Baseline PSA (ng/ml)		
* N*	34	34
Median	31.9	19.50
Range	0.17–313.2	1.97–1680

^a^One patient was included despite non-castrate levels of testosterone.

### Efficacy

**Fig. 1:**
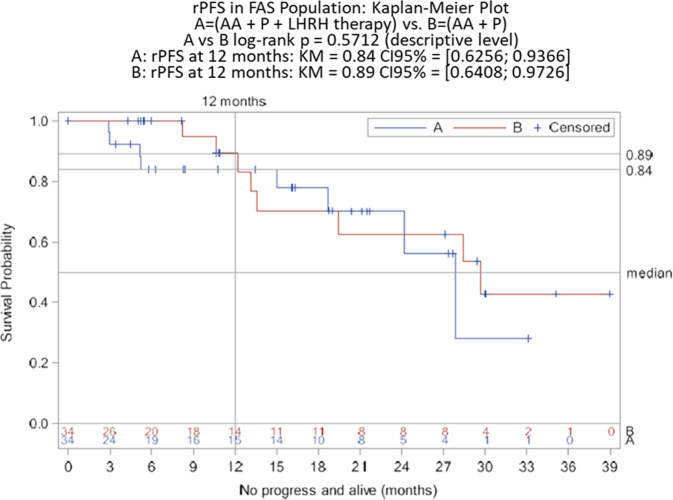
Kaplan–Meier curves of rPFS for the ITT population (FAS analysis). Results at 12 months (primary endpoint) including 95% CI for each Arm, and result of log-rank *p* test are indicated.

**Fig. 2:**
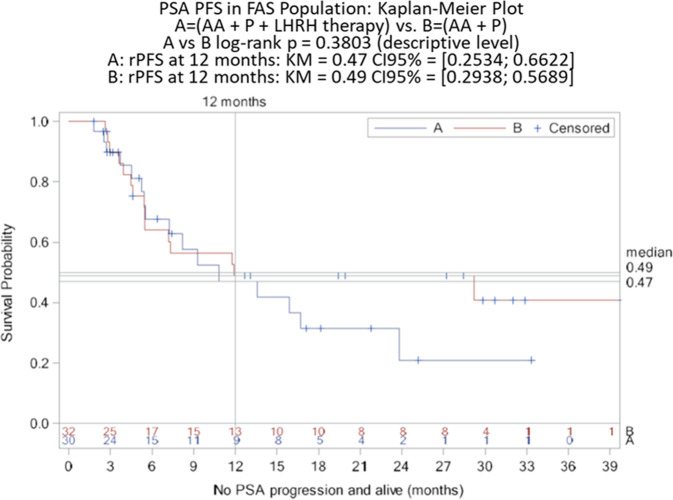
Kaplan–Meier curves of time to PSA-progression for the ITT (FAS analysis) population. Results at 12 months including 95% CI for each Arm, and result of log-rank *p* test are indicated.

The primary efficacy parameter was the rate of rPFS after 12 months and analysis was based on all patients of the ITT population (FAS analysis). According to the FAS analysis (Table 3 and Fig. 1) the rPFS rate was 0.84 in Arm A (95% CI: 0.6256–0.9366) and 0.89 in Arm B (95% CI: 0.6408–0.9726; log-rank *p* = 0.5712 at the descriptive level). PSA-response, i.e., the rate of patients achieving a post-treatment PSA decline of at least 50% according to the protocol-specific PCWG2 criteria was also calculated. According to FAS analysis, PSA response rate was 64.7% (22/34 patients) in Arm A compared to 73.5% (25/34 patients) in Arm B. Of note, 4 and 2 patients from Arm A and Arm B were not evaluable for PSA-response according to PCWG2. Time to PSA-progression was measured from the time interval from the date of randomization to the date of the PSA progression as defined in the protocol-specific PCWG2 criteria. Determination of PSA progression required that the patient would have received at least 3 cycles of therapy. According to the ITT population analysis (Table 3 and Fig. 2) the rate of PSA-progression at 12 months was 0.49 in Arm A (95% CI: 0.2534–0.6622) and 0.49 in Arm B (95% CI: 0.2938–0.6589; log-rank *p* = 0.3803 at the descriptive level).

**Table 3:** Efficacy analysis (ITT population). The data are presented as *n* (%) unless otherwise noted.

	Arm A (*N* = 34)	Arm B (*N* = 34)
rPFS at 12 months		
Rate	0.84	0.89
95% CI	0.6256–0.9366	0.6408–0.9726
*p* value (log-rank)	0.5712	
PSA response		
Rate	0.647	0.735
PSA-progression at 12 months		
Rate	0.49	0.49
95% CI	0.2534–0.6622	0.2938–0.6589
*p* value (log-rank)	0.3803	
Objective respons^a^		
Complete response	4 (20%)	3 (20%)
Partial response	4 (20%)	3 (20%)
Stable disease	8 (40%)	7 (46.7%)
Progressive disease	2 (10%)	0 (0%)
Not evaluable	2 (10%)	2 (13.3%)

^a^Based on the number of randomized patients with measurable disease at baseline (Arm A: 20 patients and Arm B: 15 patients).

Objective response was documented in the eCRF, assessed by investigators according to RECIST criteria and reviewed or corrected where necessary by the principal investigator. At baseline, objective response was documented for 20/32 patients (59%) in Arm A and for 15/34 patients (44%) in Arm B. Data regarding objective response at 12 months for all patients with measurable disease at baseline (Arm A: 20/34 patients and Arm B: 15/34 patients) are provided in Table 3. Complete response was documented for 4/20 patients (20%) in Arm A and for 3/15 patients (20%) in Arm B, partial response for 4/20 patients (20%) in Arm A and for 3/15 patients (20%) in Arm B, stable disease for 8/20 patients (40%) in Arm A and for 7/15 patients (46.7%) in Arm B, progressive disease for 2/20 patients (10%) in Arm A and for 0/15 patients (0%) in Arm B, while not evaluable were 2/20 patients (10%) in Arm A and 2/15 patients (13.3%) in Arm B.

Serum testosterone (T) levels, LH and FSH were measured centrally every 4 weeks throughout study treatment. In both arms, median serum testosterone levels decreased after initiation of study treatment to undetectable levels (<0.029 ng/ml) in all patients (Supplementary Fig. 2). In Arm A two patients were included despite non-castrate levels of testosterone (<0.5 ng/ml). LH and testosterone levels of one of the two patients reveals non-compliance with LHRH- and AA + P treatment causing the peak T level at C19 and the transient rise of mean LH-levels (C13–19), confirmed by drug accountability for AA + P. LH and FSH levels increased only in Arm B within a few cycles of study treatment after stopping LHRH-treatment Results regarding LH serum levels are provided in Supplementary Fig. 3. Although not pre-specified for analysis in the protocol, T and LH-levels of all eligible patients reveal a distinct difference throughout study treatment (Supplementary Fig. 4). Interestingly, for 6 patients in Arm B serum testosterone levels recovered (ranging from 1.59 ng/ml to 5.15 ng/ml) within 4 weeks after discontinuation of treatment with AA + P, preceded by higher-than-normal LH-levels (Supplementary Fig. 5).

### Safety evaluation

The safety population comprised all patients receiving any part of study treatment (Arm A = 32 patients and Arm B = 33 patients). Median treatment duration was comparable between the two arms (Arm A = 358 days and Arm B = 394 days).

Adverse events (grade 1–4) occurred in any patient who received any part of study treatment. Moderate and severe adverse events (grade 3–5) were reported for 94% of the patients in Arm A and for 88% of the patients in Arm B. An overview of TEAEs occurring after baseline is provided in Supplementary Table 2. Moderate and severe TEAEs (grade 3–5) were reported for 72% of patients in Arm A and for 85% of patients in Arm B. The most often reported TEAE in both arms was hypertension (Arm A: 69% of patients with grade 1–4 and 33% of patients with grade 3–5; Arm B: 64% of patients with grade 1–4 and 48% of patients with grade 3–5).

Other frequently reported TEAEs in both arms included pain (grade 1–4 only reported; Arm A: 28% and Arm B: 39%) and fatigue (grade 1–4 only reported; Arm A: 22% and Arm B: 21%). The most frequently documented TEAE based on clinical laboratory analysis was hyperglycemia (Arm A: 44% of patients with grade 1–4 and 16% of patients with grade 3–5; Arm B: 61% of patients with grade 1–4 and 18% of patients with grade 3–5).

Serious Adverse Events (SAEs) were reported for 16 patients in Arm A (23 SAEs) and for 15 patients in Arm B (19 SAEs). Relationship to study treatment was assessed by the investigator as possible for 3 SAEs occurring in 3 patients in Arm A (two events of osteonecrosis of the jaw and one event of worsening of general condition) and for 2 SAEs occurring in 2 patients in Arm B (one event of alveolitis and one event of dermatitis). Death was reported for 1 patient in Arm A (1 event of gastric cancer of severe intensity) and for 4 patients in Arm B (1 event of abdominal pain, 1 event of myocardial infarction, 1 event of acute bleeding aortic aneurism, and 1 event of worsening of general condition all of which were of severe intensity). All SAEs leading to death were assessed by the investigator as having no relationship with the study treatment.

## Discussion

The therapeutic value of LHRH-therapy continuation in mCRPC-patients remains controversial, especially since treatment with AA + P further suppresses testosterone serum levels over LHRH therapy alone. The aim of the SPARE trial was to investigate the added therapeutic value of LHRH therapy continuation in patients with asymptomatic or mildly symptomatic, chemotherapy naive mCPRC who commenced treatment AA + P.

Treatment with AA + P alone (i.e., without LHRH-therapy) resulted in high PSA response rates, a long time to PSA progression and rPFS that were comparable to those observed in the randomized, phase 3 clinical trial COU-AA-302 [9]. In addition, AA + P treatment decreased serum testosterone levels in all patients far below 0.5 ng/ml, which is the recommended castrate level [15]. Treatment with AA + P in both arms further suppressed testosterone serum levels compared to the pre-study LHRH-therapy and serum testosterone levels remained stable throughout study treatment.

Although SPARE was an exploratory study and thus no clear statistical inferences can be made, the results obtained suggest that discontinuation of LHRH-therapy may not result to decreased efficacy (by means of rPFS assessment) and therefore challenge the practice of using continuous LHRH-therapy in mCRPC patients.

Of note, the drop-out rate within the first 12 months of treatment was the same (17.6%) in both Arms so that it is unlikely that the rate of rPFS at month 12 was influenced by the dropouts. However, secondary endpoints like time to PSA progression may have been influenced by the unequal distribution and treatment duration of the drop-out patients between treatment arms.

Recently, the results from the phase 2 LACOG 0415 trial in patients with hormone-sensitive advanced/metastatic prostate cancer evaluating the use of AA + P + ADT versus apalutamide (APA) alone versus AA + P + APA were published [19]. In this trial all patients had non-castrate testosterone levels and at week 25 of treatment, the rate of patients with PSA-levels of ≤0.2 ng/ml and of radiographic progression were similar in patients receiving AA + P + APA or AA + P + ADT. In addition, testosterone serum levels decreased significantly upon treatment with AA + P + APA. However, median testosterone levels were higher in patients receiving AA + P + APA. Some patients did not reach castrate levels of testosterone (≤0.5 ng/ml) and the mean change of testosterone levels from baseline to week 25 was lower in the AA + P + APA arm compared to AA + P + ADT. The reduced efficacy of testosterone suppression may be caused, at least in part, by a compensatory increase in serum LH levels, as previously reported [20].

In contrast to the results in LACOG 0415 trial, testosterone serum levels remained undetectable in the SPARE-trial. This is in line with previous reports on maximum testosterone decline by AA + P [21]. Long-term LHRH-therapy with prolonged impairment of testicular testosterone synthesis may have affected the measured testosterone serum levels. However, the detected testosterone serum levels in all patients were far below levels that can be achieved by LHRH-therapy alone. Therefore, we conclude that suppression of testosterone serum levels in Arm B of the study was caused by the effects of abiraterone only.

A similar compensatory increase of LH and FSH levels as mentioned above within a few months after discontinuation of LHRH therapy was detected in Arm B of the SPARE trial, reflecting the clinical scenario of hypergonadotropic hypogonadism. The elevated LH and FSH levels warrant no further interventions since the suppressed testosterone level is the driver of the clinical effects. However, a rapid increase of testosterone serum levels was detected in some after discontinuation of AA + P treatment within 4 weeks, reverting the clinical scenario into hypergonadotropic hypergonadism. If the replenished testosterone serum levels are harmful to the prostate cancer patients remains controversial since bipolar testosterone treatment for mCRPC is being investigated in clinical trials with the ability to re-sensitize patients to further anti-androgen treatments [22]. However, testosterone levels and its effects should be monitored closely after discontinuation of AA + P therapy without concomitant LHRH-therapy in mCRPC patients.

Hypertension, fatigue and pain are amongst the most often reported TEAEs in line with the results from the COU-AA-302 trial [9]. Treatment with AA + P without continuation of LHRH-therapy did not increase the frequency or severity of known toxicities or caused additional or so far unknown toxicities.

In summary, based on the results of this study, discontinuation of LHRH-therapy upon treatment with AA + P in mCRPC patients can be regarded as feasible. In addition, the results do not suggest reduced efficacy of AA + P treatment alone compared to combined treatment with continuous LHRH-therapy. The results further increase the understanding of castrate- and hormone-resistant prostate cancer and provide first evidence to the current recommendation to continue LHRH-therapy, thus improving patients’ benefit during AA + P treatment. However, further confirmatory trials are required to demonstrate the efficacy of AA + P treatment without continuous LHRH-therapy in mCRPC patients.

## Supplementary information

SPARE Trial supplements

## Data Availability

All data are available upon request. The protocol can be accessed at www.clinicaltrials.gov.
