# Correction to: LHRH sparing therapy in patients with chemotherapy-naïve, mCRPC treated with abiraterone acetate plus prednisone: results of the randomized phase II SPARE trial

**DOI:** 10.1038/s41391-023-00682-2

**Published:** 2023-05-30

**Authors:** Carsten-Henning Ohlmann, Michelle Jäschke, Peter Jaehnig, Susanne Krege, Jürgen Gschwend, Heidrun Rexer, Kerstin Junker, Roger Zillmann, Christoph Rüssel, Eva Hellmis, Henrik Suttmann, Martin Janssen, Jan Marin, Andreas Hübner, Michael Mathers, Jochen Gleißner, Michael Scheffler, Susan Feyerabend, Jens Telle, Jörg Klier, Michael Stöckle

**Affiliations:** 1 Department of Urology, Saarland University Medical Center, Homburg/Saar, Germany; 2 Department of Urology, Johanniter-Kliniken Bonn, Bonn, Germany; 3 pj statistics, Berlin, Germany; 4 Department of Urology, Evangelische Kliniken Essen Mitte, Essen, Germany; 5 Department of Urology, Klinikum Rechts der Isar, Technical University Munich, Munich, Germany; 6 Meckevidence, Schwarz, Germany; 7 Urologie Pankow, Berlin, Germany; 8 Urologie Borken, Borken, Germany; 9 Urologicum-Duisburg Fachärztesozietät, Duisburg, Germany; 10 Urologikum Hamburg MVW, Hamburg, Germany; 11 Department of Urology, University Münster, Münster, Germany; 12 Urologie Kempen, Münster, Germany; 13 Zentrum für Onkologie und Urologie, Rostock, Germany; 14 Pandamed, Remscheid, Germany; 15 Uro-Gyn-Praxis Am Wall, Wuppertal, Germany; 16 Urologische Gemeinschaftspraxis, Zwickau, Germany; 17 Studienpraxis Urologie, Nürtingen, Germany; 18 Urologische Praxisgemeinschaft, Wolfsburg, Germany; 19 Urologische Partnerschaft, Köln, Germany

**Keywords:** Prostate cancer, Cancer therapy

Correction to: *Prostate Cancer and Prostatic Diseases* 10.1038/s41391-022-00533-6, published online 16 April 2022

In author contribution by MJ was given incorrectly as “Patient data and study materials, Manuscript editing and review”

but it should have been “Patient data and study materials, data analysis and interpretation, Manuscript editing and review.”

The original article has been corrected.

