# Supplementary material for: LHRH sparing therapy in patients with chemotherapy-naïve, mCRPC treated with abiraterone acetate plus prednisone: results of the randomized phase II SPARE trial
Source: Prostate Cancer Prostatic Dis. 2022 Apr 16;25(4):778–84. doi: 10.1038/s41391-022-00533-6 (PMC9705242; doi:10.1038/s41391-022-00533-6)
Supplement: Supplementary file 1 — SPARE Trial supplements [file 41391_2022_533_MOESM1_ESM.docx]

**SPARE Publication – Supplements**

1. **Figure 1:** Consort diagram
2. **Figure 2:** Serum testosterone levels all patients
3. **Figure 3:** Serum LH levels all patients
4. **Figure 4:** Serum testosterone and LH levels eligible patients
5. **Figure 5:** Testosterone and LH levels of patients from Arm B with rapid testosterone recovery
6. **Table 1:** Inclusion and exclusion criteria.


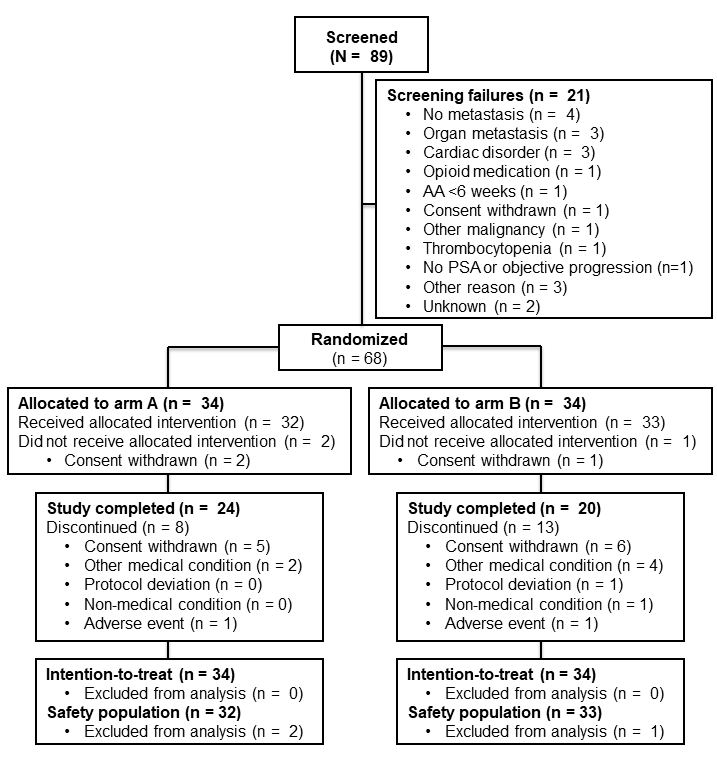


**Supplemental** **Fig. 1.** CONSORT diagram. According to the protocol, the intention-to-treat population and the safety populations were to be analyzed. AA=Abiraterone acetate. P=prednisone.


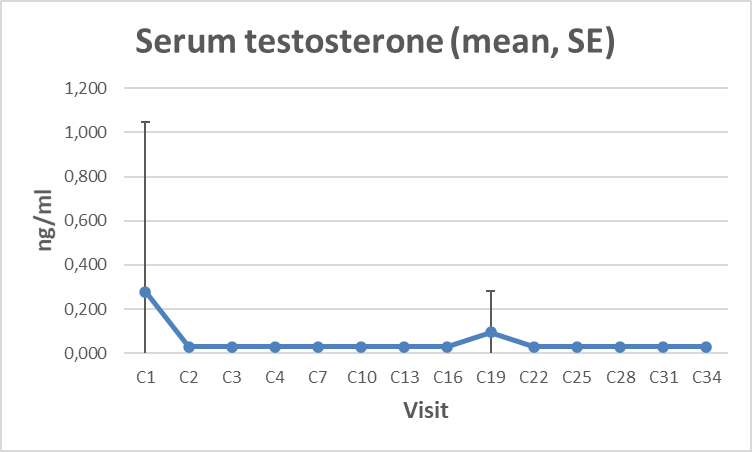


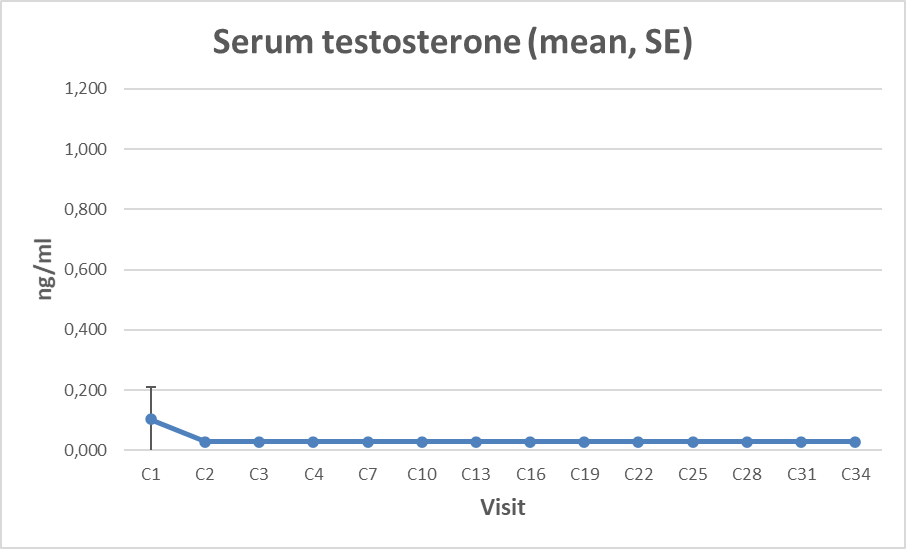


**Supplemental** **Fig. 2.** Serum testosterone levels at different study visits for all patients with available data in Arm A (upper panel, n = 28) and Arm B (lower panel, n = 31).

**
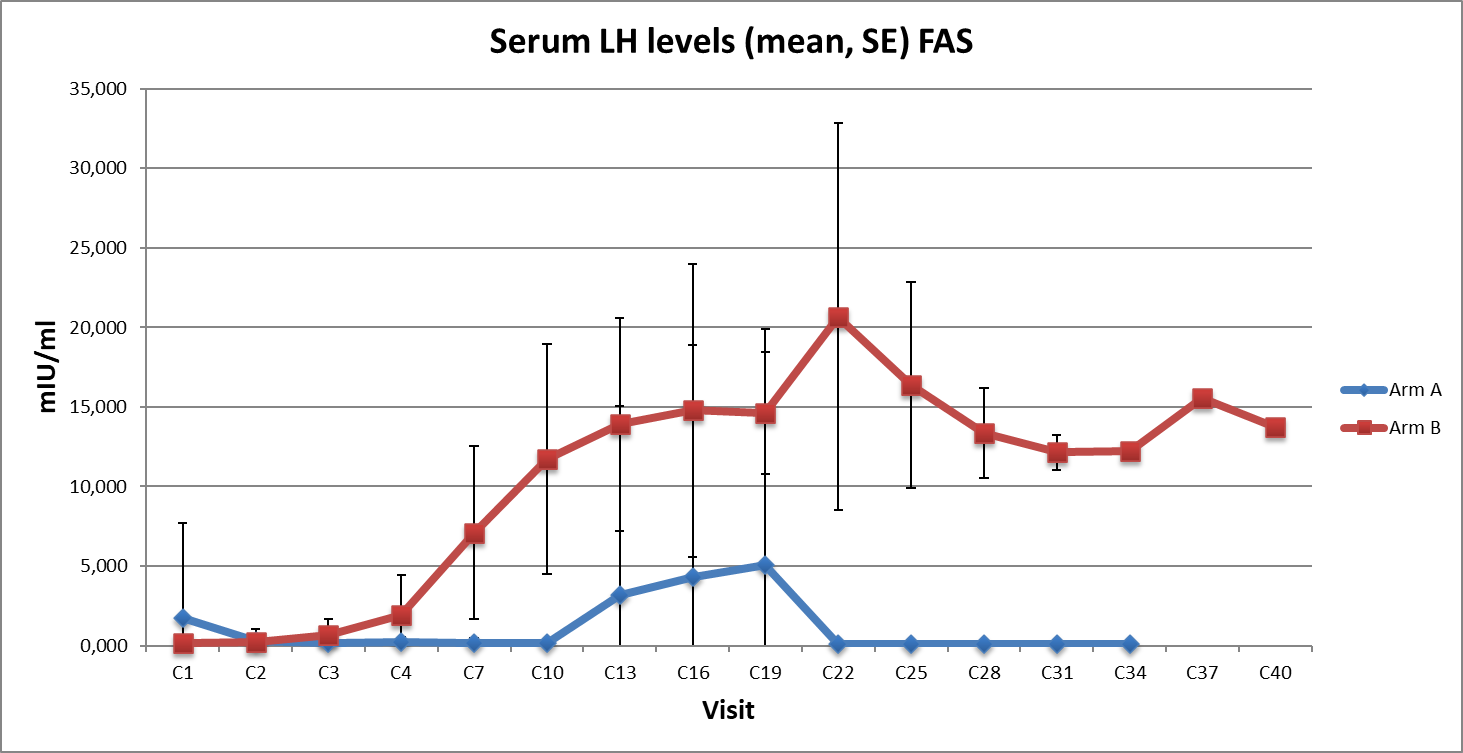
**

**Supplemental** **Fig. 3.** Serum LH levels at different study visits for all patients with available data (FAS) in Arm A (n = 28) and Arm B (n = 31).


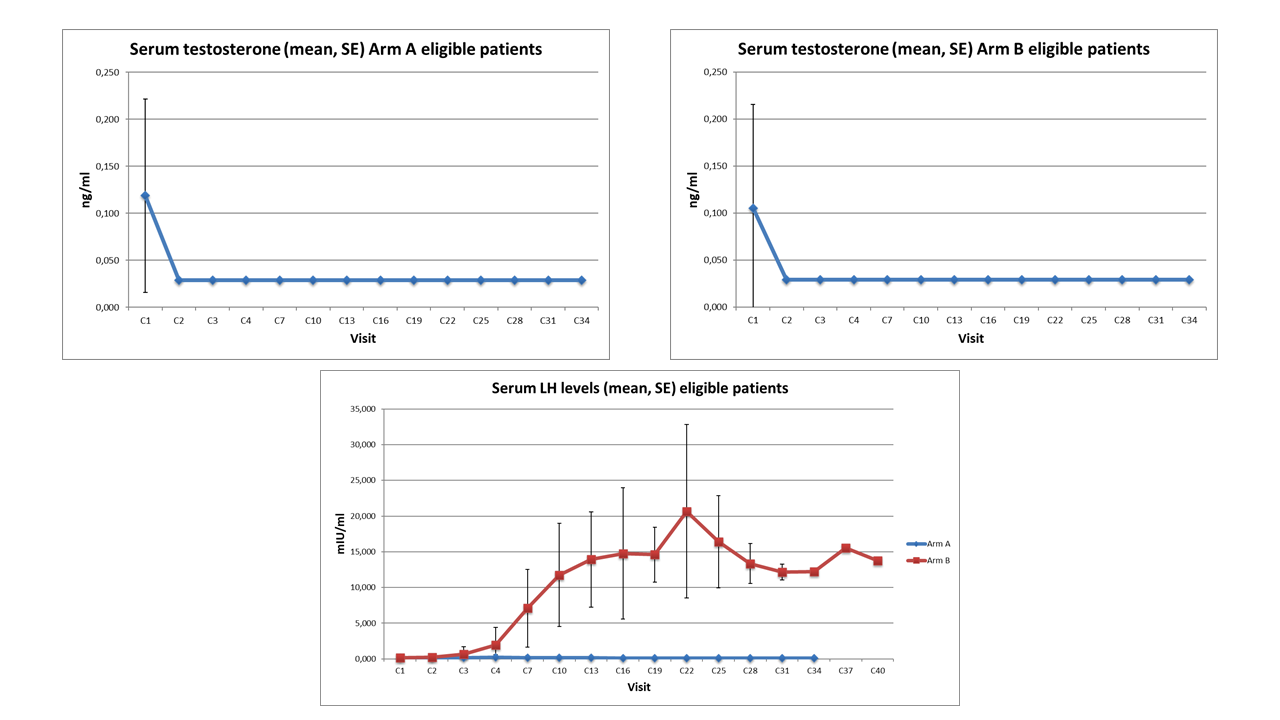


**Supplemental** **Fig. 4.** Serum T and LH levels at different study visits for all eligible patients with available data in Arm A (n = 25) and Arm B (n = 25).


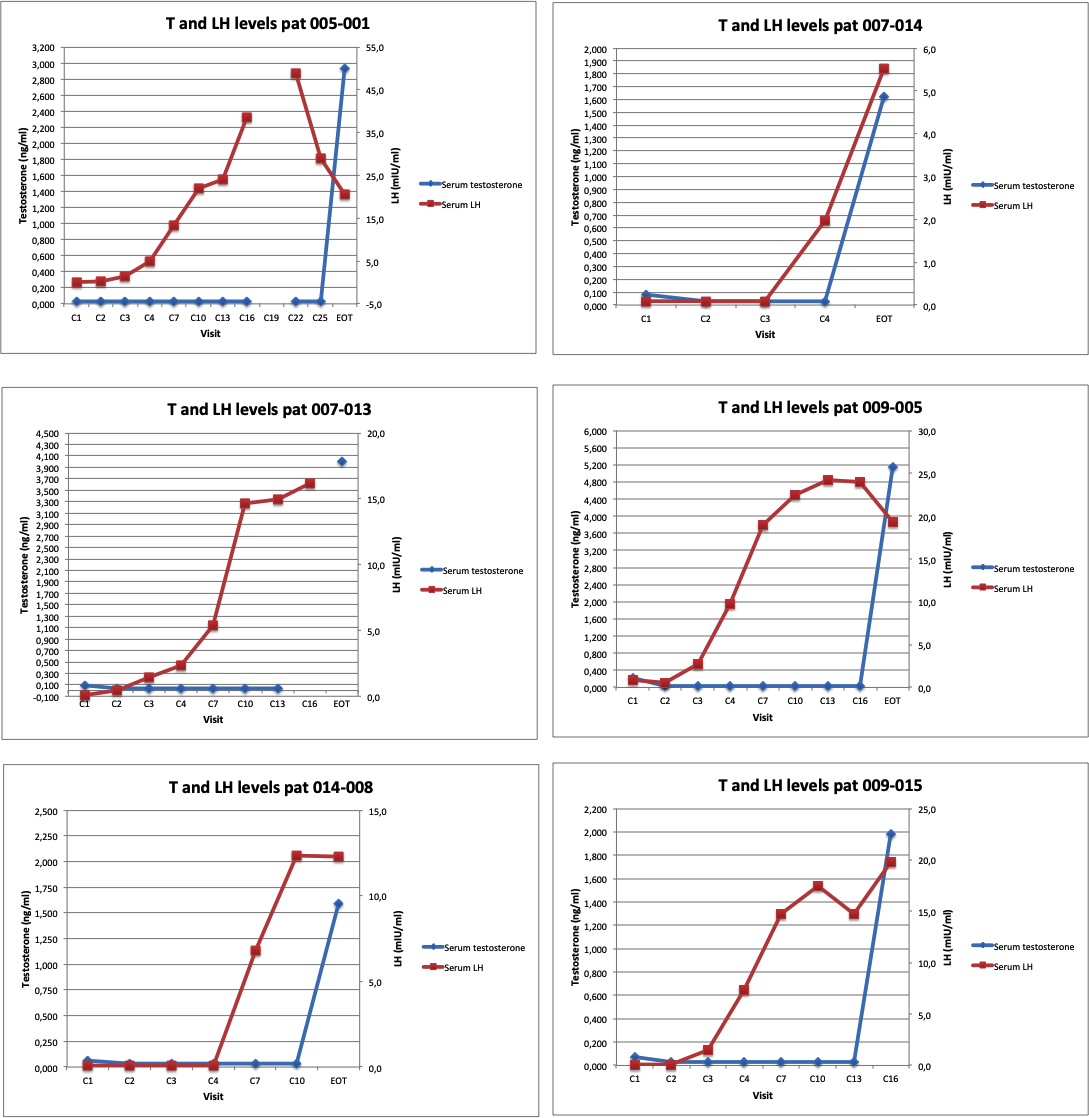


**Supplemental** **Fig. 5.** Testosterone and LH levels of six patients from Arm B with rapid testosterone recovery after cessation of study medication**.**

| **Inclusion criteria** |
| --- |
| 1. Male aged 18 years and above 2. Histologically or cytologically confirmed adenocarcinoma of the prostate 3. Metastatic disease documented by positive CT/MRI and/or bone scan. If lymph node metastasis is the only evidence of metastasis, it must be ≥2 cm in diameter 4. Prostate cancer progression documented by PSA according to PCWG2 or radiographic progression according to modified RECIST criteria 5. Asymptomatic or mildly symptomatic from prostate cancer. A score of 0-1 for the question of worst pain within last 24 hours will be considered asymptomatic, and a score of 2-3 will be considered mildly symptomatic. 6. Medically castrated, with testosterone levels of <20-50 ng/dl (< 2.0 nM). 7. Combined androgen blockade is permitted, but not required. If patients received combined androgen blockade with an anti-androgen they must have shown PSA progression after discontinuing the anti-androgen prior to enrolment (≥4 weeks since last flutamide, ≥6 weeks since last bicalutamide or nilutamide). |
| **Exclusion criteria** |
| 1. Surgical castration (i.e. orchiectomy). 2. Application of any LHRH-therapy (LHRH-analogue or LHRH-antagonist) within 3months (for patients receiving a 3-months formulation) or 1 months (for patients receiving a 1-month formulation) prior to Cycle 1 day 1. 3. Patients receiving a 6- or 12-months formulation of LHRH-therapy 4. Active infection or other medical condition that would make prednisone/prednisolone (corticosteroid) use contraindicated 5. Any chronic medical condition requiring a higher dose of corticosteroid than 5mg prednisone/prednisolone bid. 6. Pathological finding consistent with small cell carcinoma of the prostate 7. Liver or visceral organ metastasis 8. Known brain metastasis 9. Use of opiate analgesics for cancer-related pain, including codeine, tramadol, tilidin and others, currently or anytime within 4 weeks of Cycle 1 Day 1. 10. Prior cytotoxic chemotherapy or biologic therapy for the treatment of CRPC 11. Radiation therapy for treatment of the primary tumour within 6 weeks of Cycle 1, Day 1 12. Radiation or radionuclide therapy for treatment of metastatic CRPC 13. Prior treatment with abiraterone acetate or other CYP17 inhibitors (ketoconazole, TAK700, TOK001), enzalutamide (Xtandi) or investigational agents targeting the androgen receptor for prostate cancer for more than 7 days 14. Prior systemic treatment with an azole drug (e.g. fluconazole, itraconazole) within 4 weeks of Cycle 1, Day 1 15. Prior flutamide (Eulexin) treatment within 4 weeks of Cycle 1, Day 1 (patients whose PSA did not decline for three or more months in response to antiandrogen given as a second line or later intervention will require only a two-week washout prior to Cycle 1, Day 1) 16. Bicalutamide (Casodex), nilutamide (Nilandron) within 6 weeks of Cycle 1 Day 1 (patients whose PSA did not decline for three or more months in response to antiandrogen given as a second line or later intervention will require only a two-week washout prior to Cycle 1, Day 1) 17. Uncontrolled hypertension (systolic BP ≥160 mmHg or diastolic BP ≥95 mmHg). Patients with a history of hypertension are allowed provided that blood pressure is controlled by anti-hypertensive treatment 18. Active or symptomatic viral hepatitis or chronic liver disease 19. History of pituitary or adrenal dysfunction 20. Clinically significant heart disease as evidenced by myocardial infarction, or arterial thrombotic events in the past 6 months, severe or unstable angina, or New York Heart Association (NYHA) Class II-IV heart disease or cardiac ejection fraction measurement of <50% at baseline |

**Supplemental Table 1**. Inclusion and exclusion criteria.

|  | Arm A (*N* = 32) | | Arm B  (*N* = 33) | |
| --- | --- | --- | --- | --- |
|  | Grade 1-4 | Grade 3-5 | Grade 1-4 | Grade 3-5 |
| AEs | 32 (100) | 30 (94) | 33 (100) | 29 (88) |
| TEAEs | 31 (97) | 23 (72) | 33 (100) | 28 (85) |
| Hypertension | 22 (69) | 12 (38) | 21 (64) | 16 (48) |
| Elevated cholesterol | 10 (31) | 0 (0) | 13 (36) | 0 (0) |
| Anemia | 5 (16) | 1 (3) | 14 (42) | 0 (0) |
| Hyperglycemia | 14 (44) | 5 (16) | 20 (61) | 6 (18) |
| Elevated triglycerides | 13 (41) | 0 (0) | 16 (48) | 0 (0) |
| Elevated AP | 12 (38) | 1 (3) | 10 (30) | 0 (0) |
| Elevated ALT | 10 (31) | 2 (6) | 12 (36) | 2 (6) |
| Elevated AST | 8 (25) | 0 (0) | 7 (21) | 1 (3) |
| Hypokalemia | 9 (28) | 4 (12) | 5 (15) | 0 (0) |
| Elevated GGT | 5 (16) | 3 (9) | 4 (12) | 2 (6) |
| Elevated creatinine | 5 (16) | 0 (0) | 10 (30) | 0 (0) |
| Pain | 9 (28) | 0 (0) | 13 (39) | 2 (6) |
| Fatigue | 7 (22) | 0 (0) | 7 (21) | 0 (0) |
| Hematuria | 3 (9) | 1 (3) | 4 (12) | 0 (0) |
| Bronchial infection | 4 (13) | 0 (0) | 5 (15) | 0 (0) |
| Osteonecrosis of jaw | 4 (13) | 2 (6) | 2 (6) | 1 (3) |
| Skin disorder | 4 (13) | 0 (0) | 8 (27) | 1 (3) |
| Hot flashes | 1 (3) | 0 (0) | 4 (12) | 0 (0) |
| Edema | 1 (3) | 0 (0) | 6 (18) | 2 (6) |
| Loss of appetite | 0 (0) | 0 (0) | 4 (12) | 0 (0) |
| Hematoma | 0 (0) | 0 (0) | 4 (12) | 0 (0) |

**Supplemental Table 2**. Treatment emergent adverse events (TEAEs) occurring after baseline within any of the two Arms of the study (Safety population). The data are presented as n (%).

AP = alkaline phosphatase, ALT = alanine aminotransferase, AST = aspartate aminotransferase, GGT = gamma-glutamyl transferase
